# Design and Production of a Recombinant Hybrid Toxin to Raise Protective Antibodies against *Loxosceles* Spider Venom

**DOI:** 10.3390/toxins11020108

**Published:** 2019-02-12

**Authors:** Paula A. L. Calabria, Lhiri Hanna A. L. Shimokawa-Falcão, Monica Colombini, Ana M. Moura-da-Silva, Katia C. Barbaro, Eliana L. Faquim-Mauro, Geraldo S. Magalhaes

**Affiliations:** Laboratory of Immunopathology, Butantan Institute, São Paulo 05503-900, Brazil; paula.calabria@butantan.gov.br (P.A.L.C.); lhiri.hanna@gmail.com (L.H.A.L.S.-F.); monica.colombini@butantan.gov.br (M.C.); ana.moura@butantan.gov.br (A.M.M.-d.-S.); katia.barbaro@butantan.gov.br (K.C.B.); eliana.faquim@butantan.gov.br (E.L.F.-M.)

**Keywords:** phospholipases D, metalloproteases, *Loxosceles* spp., recombinant toxins, hybrid immunogen, neutralizing antibodies, antivenoms

## Abstract

Human accidents with spiders of the genus *Loxosceles* are an important health problem affecting thousands of people worldwide. Patients evolve to severe local injuries and, in many cases, to systemic disturbances as acute renal failure, in which cases antivenoms are considered to be the most effective treatment. However, for antivenom production, the extraction of the venom used in the immunization process is laborious and the yield is very low. Thus, many groups have been exploring the use of recombinant *Loxosceles* toxins, particularly phospholipases D (PLDs), to produce the antivenom. Nonetheless, some important venom activities are not neutralized by anti-PLD antibodies. Astacin-like metalloproteases (ALMPs) are the second most expressed toxin acting on the extracellular matrix, indicating the importance of its inclusion in the antigen’s formulation to provide a better antivenom. Here we show the construction of a hybrid recombinant immunogen, called LgRec1ALP1, composed of hydrophilic regions of the PLD and the ALMP toxins from *Loxosceles gaucho*. Although the LgRec1ALP1 was expressed as inclusion bodies, it resulted in good yields and it was effective to produce neutralizing antibodies in mice. The antiserum neutralized fibrinogenolytic, platelet aggregation and dermonecrotic activities elicited by *L. gaucho*, *L. laeta*, and *L. intermedia* venoms, indicating that the hybrid recombinant antigen may be a valuable source for the production of protective antibodies against *Loxosceles* ssp. venoms. In addition, the hybrid recombinant toxin approach may enrich and expand the alternative antigens for antisera production for other venoms.

## 1. Introduction

In view of the wide geographical distribution, the large number of individuals affected and the evolution of the clinical picture, the accidents with spiders of the genus *Loxosceles*, denominated loxoscelism, have received great attention from public health [1,2,3]. In Brazil, most of the human accidents are related to three main *Loxosceles* species: *Loxosceles gaucho*, *Loxosceles intermedia*, and *Loxosceles laeta* [3,4]. The loxoscelism is associated with a number of clinical symptoms including edema, an intense inflammatory reaction at the site of the bite, which can progress to a typical necrotic lesion on the skin with gravitational scattering, known as cutaneous loxoscelism [3,5,6,7]. In rare cases, cutaneous loxoscelism may progress to systemic manifestations (cutaneous-visceral loxoscelism) and the symptoms of this clinical condition usually begin 24 h after the spider bites, which is characterized by anemia, jaundice, intravascular hemolysis, platelet aggregation, and, in more severe cases, renal failure [8].

The venom of *Loxosceles* spp. is composed of numerous protein molecules with toxic and/or enzymatic activity [2,3,8,9,10,11], such as phospholipases D, metalloproteases, serine proteases, hyaluronidases, allergens, serine protease inhibitors, and peptides classified as cystine knot inhibitors [9,12,13,14,15,16]. Studies have shown that phospholipases D (PLDs) are the most abundant toxins able to elicit a cascade of adverse pharmacological events such as inflammation [13,17] dermonecrosis [11,13,18,19,20,21], platelet aggregation [21,22,23], hemolysis [13,23,24], and nephrotoxicity [25,26], among others.

Currently, the treatment used for human envenoming includes the use of anti-arachnid serum that in Brazil is obtained by immunizing horses with a mixture of venoms from *Loxosceles gaucho*, *Phoneutria nigriventer* spiders and the scorpion *Tityus serrulatus* (SAAr) or the use of anti-loxoscelic serum that is obtained with the mixture of *L. intermedia, L. laeta*, and *L. gaucho* venoms (SALox), usually associated with corticosteroids [1,27,28,29,30,31,32]. However, the extraction of the amount of venom needed for horse immunizations is expensive, laborious, and the yield obtained is very low. This fact has led some researchers to use recombinant toxins such as the PLDs [33,34,35,36] or even peptides from these toxins [30,37,38] to obtain the antiserum. Nonetheless, the antiserum obtained in this way is specific to PLD and did not neutralize all venom activities due to the synergistic action of other toxins that contribute to the deleterious effects of the venom [6,8].

In this sense, studies have shown that the astacin-like metalloproteases (ALMPs) are the second most abundant class of toxins in the venom glands of *L. laeta* [39] and *L. intermedia* [15] and appear to contribute to the envenomation picture since they hydrolyze some components of the extracellular matrix such as collagen [40], fibronectin [9,41,42], and fibrinogen [9,41,43,44,45]. Therefore, considering that the PLDs and ALMPs are the main toxins present in the venom of *Loxosceles* spp., in this work, we envisaged the construction of a hybrid recombinant toxin composed of the hydrophilic regions of a PLD and ALMP from *L. gaucho* to raise neutralizing antibodies in mice against of the venom of the three predominant *Loxosceles* spp. spiders that cause envenomation in Brazil. Therefore, this hybrid molecule might be an interesting tool to enhance and/or expand the possibilities to raise protective antiserum against *Loxosceles* spp. venom and this approach may also be applied to other venoms.

## 2. Results

### 2.1. Construction of the Hybrid Molecule LgRec1ALP1

**Figure 1:**
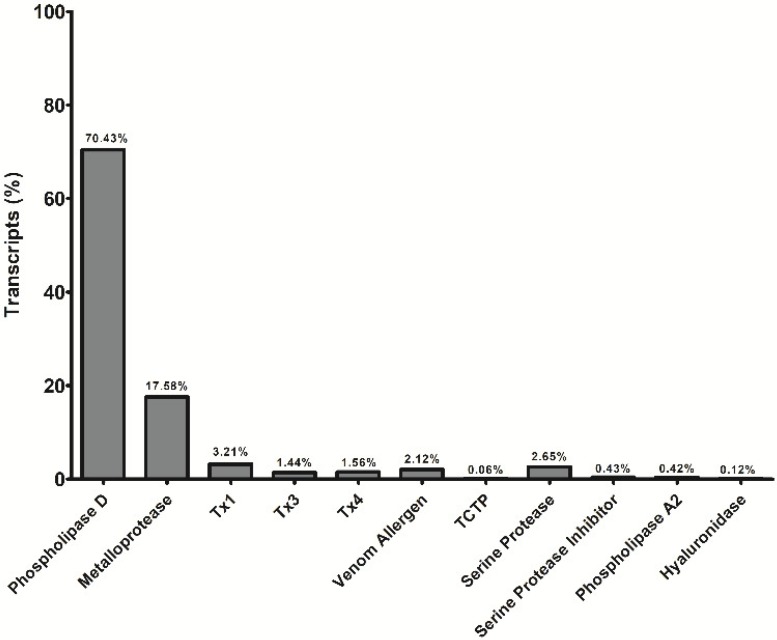
Graph showing the percentage of toxin transcripts in relation to the total toxins’ transcripts found in *L. gaucho* venom gland. TX (similar to insecticide toxin); TCTP (similar to tumor-controlled translation protein).

In order to know the main toxin transcripts present in the venom gland of *Loxosceles gaucho*, a transcriptomic approach was performed and the analysis showed that 22.36% of all sequences gave match to toxins already described in the database. Among them, it was observed that phospholipase D (PLD) and astacin-like metalloprotease (ALMPs) were the most abundant, corresponding to 70.43 and 17.58%, respectively (Figure 1). Taking into consideration this result and the important activities of these toxins in the venom, they were chosen to make part of a hybrid immunogen construction.

Analyzing all the PLDs transcripts with identity greater than 97%, it was observed that the largest group contained 37% of all PLDs sequences, and a PLD called LgRec1 [20], present in this group was chosen to be part of the hybrid immunogen. Among the metalloprotease’s transcripts with identity greater than 95%, the largest group contained 45% of all metalloproteinase transcripts, and a sequence called LgALP1 was selected from this group to be part of the hybrid immunogen.

**Figure 2:**
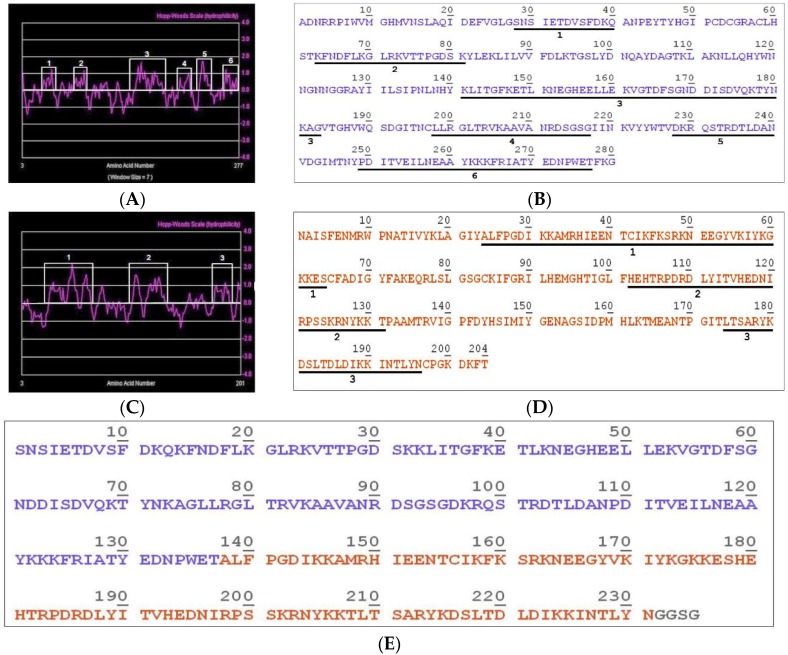
Sequence analysis of phospholipase D LgRec1 and metalloprotease LgALP1 to construct the hybrid immunogen LgRec1ALP1. Hydrophilicity plots of LgRec1 (**A**) and LgALP1 (**C**), deduced by the ProtScale program with Hopp–Woods scale, where the hydrophilic regions of each molecule are indicated with boxes. Sequence of LgRec1 (**B**) and LgALP1 (**D**) showing the predicted hydrophilic amino acids numbered and underlined. (**E**) Amino acid sequence of the hybrid immunogen LgRec1ALP1 containing only the hydrophilic regions of phospholipase D (PLD) LgRec1 (blue) and astacin-like metalloprotease (ALMP) LgALP1 (orange). Sequence numbers correspond to amino acid positions in the sequence.

The sequences of the two toxins were submitted to the ProtScale Tool program using the Hopp-Woods scale [46] to identify the hydrophilic regions of the molecules. This scale performs the prediction of potentially antigenic regions of polypeptides, where values greater than 0 are hydrophilic. After analysis, six and three hydrophilic peaks were found in the PLD LgRec1 (Figure 2A) and the metalloprotease LgALP1 sequences (Figure 2C), respectively.

The hydrophilic peptides found in the PLD LgRec1 (Figure 2B) and the ALMP LgALP1 (Figure 2D) are numbered and underlined. These peptides were then joined to form the hybrid immunogen that was called LgRec1ALP1 (Figure 2E). The exposures of these peptides on the surface of each toxin were also analyzed on the predicted tridimensional structure of the PLD LgRec1 (Figure S1) and the ALMP LgALP1 (Figure S2). To predict these structures, the crystal of a phospholipase D (3LRH) from *L. intermedia* [47] and the metalloprotease (3LQ0) from *Astacus astacus* [48] were used as templates by Phyre2 program. Since most of the hydrophilic peptides ended or stated in random coils, which are flexible loops from the original proteins, no linkers were used in the construction. In addition, to analyze the identity of the hydrophilic peptides with other spiders PLDs and metalloproteases, they were aligned against the PLDs LiRecDT1 (ABA62021) and Smase I (AAM21154) from *L. intermedia* and *L. laeta*, respectively, as well as with the metalloproteases LALP2 (ACV52010) and LLAE0237C (EY188609), also from *L. intermedia* and *L. laeta*, respectively. As can be seen, the peptides show higher identity with the PLDs (Table 1) and the metalloproteases (Table 2) from *L. intermedia*.

### 2.2. Expression and Purification of the Hybrid Immunogen LgRec1ALP1

**Figure 3:**
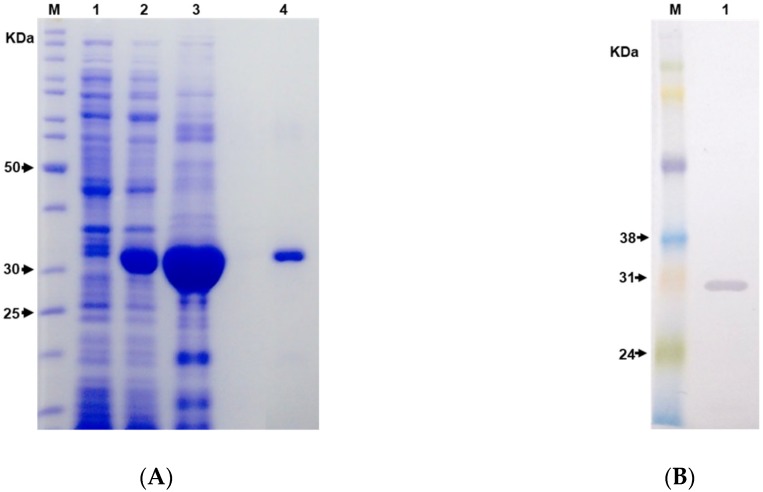
Analysis of the recombinant hybrid immunogen LgRec1ALP1. Numbers on the left correspond to the position of molecular mass markers in kDa (M). (**A**) 12.5% sodium dodecyl sulfate–polyacrylamide gel electrophoresis (SDS-PAGE) gel showing expression and purification of the hybrid immunogen LgRec1ALP1 overexpressed in *E. coli* BL21 Star™ (DE3) at 30 °C. Protein was visualized on a 12.5% SDS/polyacrylamide gel under reducing conditions and stained with Coomassie blue. 1 and 2: Extract from BL21 Star™ (DE3) before and after isopropyl-β-d-thiogalactoside (IPTG) (1 mM) induction, respectively; 3: Bacterial pellet lysed by sonication; 4: LgRec1ALP1 solubilized in urea 6M and purified by IMAC. (**B**) Western blot analysis. 1: Purified LgRec1ALP1 was separated by 12.5% SDS-PAGE, transferred onto nitrocellulose membrane, incubated with monoclonal anti-polyhistidine antibody and revealed with 4-chloro-1-naphthol.

The sequence of the hybrid immunogen LgRec1Alp1 was cloned into pET28a+ vector, transformed into chemically competent *E. coli* strain BL21 Star™ (DE3) and expressed at 30 °C for 4 h under induction of 1 mM of isopropyl-β-d-thiogalactoside (IPTG). The sodium dodecyl sulfate–polyacrylamide gel electrophoresis (SDS-PAGE) analysis (Figure 3A) indicates that the LgRec1Alp1 was successfully expressed as shown by the presence of an expected band with a molecular mass around 30 kDa after IPTG induction (lane 2). However, LgRec1Alp1 was expressed in the insoluble form, since after cell sonication the protein could only be seen in the pellet of cell lysed (lane 3). Therefore, the pellet was solubilized in 6 M urea and purified by immobilized metal affinity chromatography (IMAC) taking advantage of the 6xHis tag present at the C-terminus of LgRec1ALP1. After dialysis to remove urea, white clumps were observed in the dialysis bag, indicating that the recombinant LgRec1ALP1 was insoluble (lane 4). The purified LgRec1ALP1 was then subjected to identification by Western blot using an anti-His tag monoclonal antibodies (Figure 3B). The average yield of LgRec1ALP1 was 3.5 mg per liter of cell culture.

### 2.3. Immunogenicity and Cross-Reactivity of Anti-LgRec1ALP1

**Figure 4:**
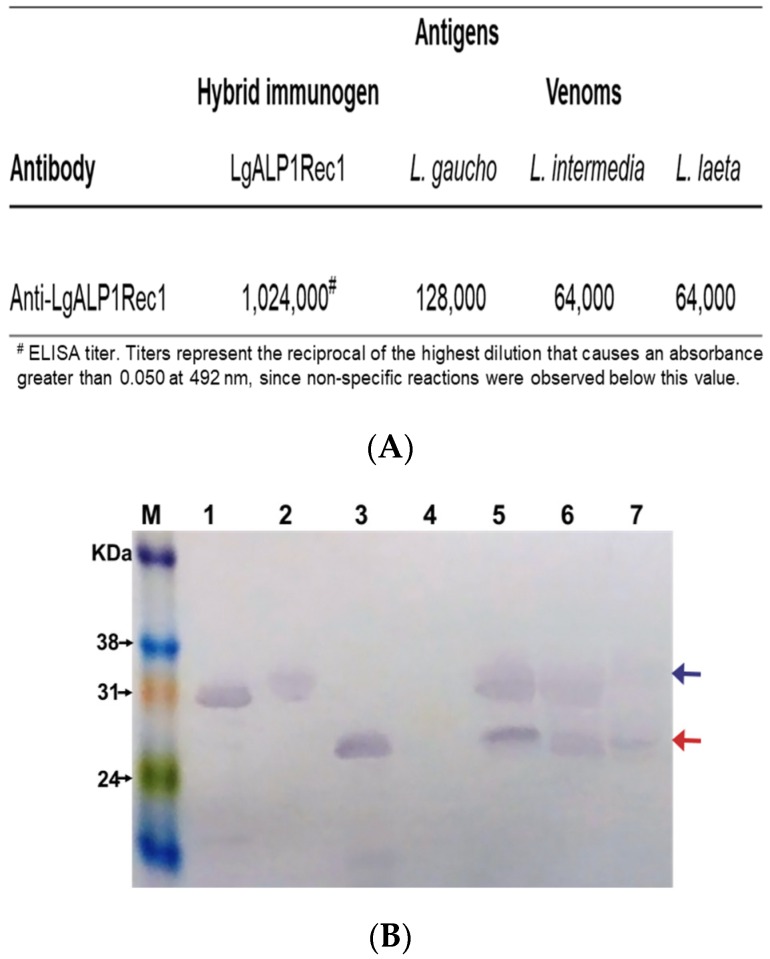
Evaluation of anti-LgRec1ALP1 by ELISA and Western blot. (**A**) Titration of LgRec1ALP1 antibodies by ELISA. The plates were coated with 5 μg/mL of the hybrid immunogen LgRec1ALP1, *L. gaucho*, *L. intermedia*, or *L. laeta* venoms. The absorbances of the samples were determined at 492 nm. (**B**) Recognition of anti-LgRec1ALP1 by Western blot. Proteins were separated by SDS-PAGE, transferred onto nitrocellulose membrane and incubated with anti-LgRec1ALP1. Numbers on the left correspond to the position of molecular mass markers (M). Recombinant hybrid immunogen LgRec1ALP1 (1); Recombinant PLD LgRec1 (2); Recombinant ALMP LgALP1 (3); Nonrelated recombinant protein EGFP (4); *L. gaucho* venom (5); *L. intermedia* venom (6); *L. laeta* venom (7). Blue and red arrows indicate the position for PLDs and ALMPs, respectively.

After dialysis, the recombinant LgRec1ALP1 in its colloidal state was mixed with Montanide and used via subcutaneous injection to produce polyclonal antibodies in mice. The immunoglobulins contained in the antiserum were then purified by Hi-Trap protein G affinity column. The titer of purified IgGs anti-LgRec1ALP1 was determined by ELISA using the recombinant LgRec1ALP1, *L. gaucho*, *L. laeta*, and *L. intermedia* venoms as coating antigens. The result shows, as expected, a higher titer for the recombinant LgRec1ALP1, followed by *L. gaucho*, *L. laeta*, and *L. intermedia* venoms (Figure 4A).

The specificity of the antibodies was also evaluated by Western blot using the recombinant PLD LgRec1 and the recombinant metalloprotease LgALP1 and the venoms of *L. gaucho*, *L. intermedia*, and *L. laeta*. The recombinant enhanced green fluorescent protein (EGFP) was used as non-related protein control. The result shows that the anti-LgRec1ALP1 recognized all recombinant toxins as well as the bands with approximate molecular mass expected for phospholipases D (blue arrow) and metalloproteases (red arrow) in all venoms. However, the bands corresponding to phospholipase D showed lower intensity in the *L. laeta* venom (Figure 4B).

### 2.4. Neutralization Assays

#### 2.4.1. Neutralization of Fibrinogen Degradation Caused by *Loxosceles* spp. Venoms

**Figure 5:**
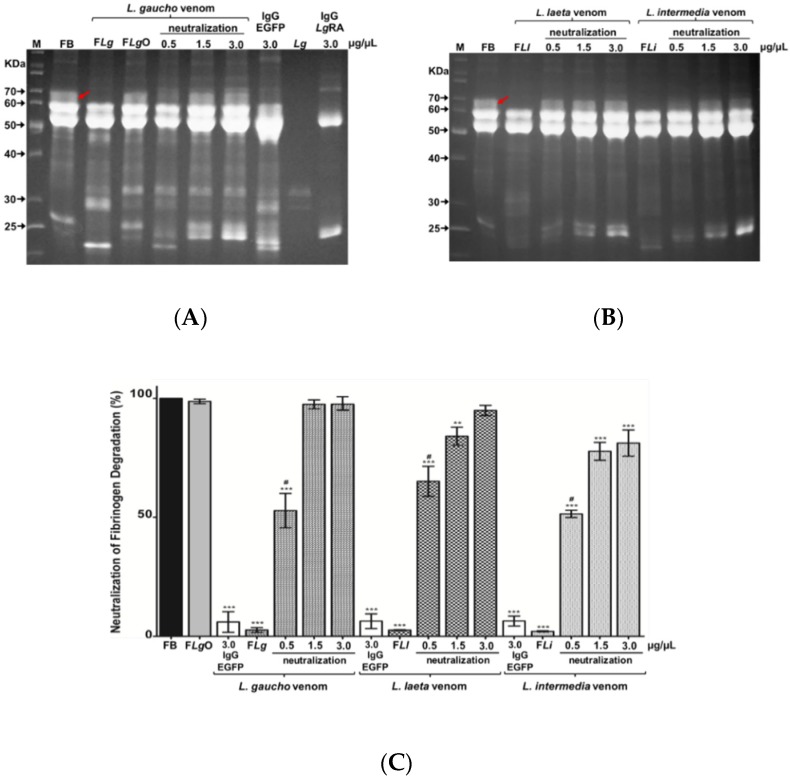
Evaluation of neutralization fibrinogen degradation (α subunit) by anti-LgRec1ALP1. Fibrinogen (FB) was incubated with 0.15 μg/µL of *L. gaucho* (F*Lg*), *L. laeta* (F*Ll*), or *L. intermedia* (F*Li*) venoms previously incubated or not with 0.5, 1.5, or 3.0 μg/µL of anti-LgRec1ALP1 (neutralization). Nonrelated IgG anti-EGFP (IgG-EGFP) pre-incubated with *L. gaucho*, *L. laeta*, or *L. intermedia* venoms were used as negative controls. FB: fibrinogen in Tris-HCl buffer; F*Lg*O: fibrinogen incubated with *L. gaucho* venom and 1,10-phenanthroline (10 mM); IgG-LgRA: IgG anti-LgRec1ALP1. (**A**,**B**) SDS-PAGE gels showing the neutralization of fibrinogen α subunit (red arrow) degradation by anti-LgRec1ALP1. Samples were visualized on a 12% SDS/polyacrylamide gel under reducing conditions and stained with Coomassie blue. Numbers on the left correspond to the position of molecular mass markers (M) in kDa. (**C**) Graph showing the quantification of degradation of fibrinogen α subunit from SDS-PAGE analyzed by ImageJ densitometry software. Values given are the average ± SEM (*n* = 3). Significance was evaluated with an ANOVA one-way with the post-hoc Tukey test; (**) indicates *p* < 0.01, (***) indicates *p* < 0.001. # indicates statistical significance with *p* < 0.001 between samples of the venom’s groups.

To evaluate the ability of anti-LgRec1ALP1 to neutralize the proteolytic action of metalloproteases, the venoms of *L. gaucho*, *L. laeta*, and *L. intermedia* were pre-incubated with anti-LgRec1ALP1 and this mixture was then incubated with bovine fibrinogen. The samples were applied to SDS-PAGE (Figure 5A,B) and the percentage of neutralization of fibrinogen alpha chain degradation was evaluated with the ImageJ program (Figure 5C). The data show that 1.5 and 3.0 μg/µL of anti-LgRec1ALP1 was able to completely neutralize the degradation of the α subunit of fibrinogen (Figure 5A,B red arrows) caused by the *L. gaucho* venom (Figure 5B), 85–95% for *L. laeta* venom and 78–83% for *L. intermedia* venom (Figure 5C).

The role of metalloproteinases in the fibrinogen alpha subunit degradation was confirmed by incubating *L. gaucho* venom with the Zn^2+^ chelating metalloprotease inhibitor 1,10-phenanthroline (F*Lg*O), which completely abolished the degradation (Figure 5A,C). As a negative control, *L. gaucho*, *L. laeta*, and *L. intermedia* venoms were pre-incubated with a nonrelated IgG (anti-EGFP), which showed no neutralization activity (Figure 5C), represented by lane IgG EGFP in Figure 5A, where we can also visualize the bands related to *L. gaucho* venom (*Lg*) and IgG anti-LgRec1ALP1 (*Lg*RA).

#### 2.4.2. Neutralization of Platelets Aggregation Caused by *Loxosceles* spp. Venoms

**Figure 6:**
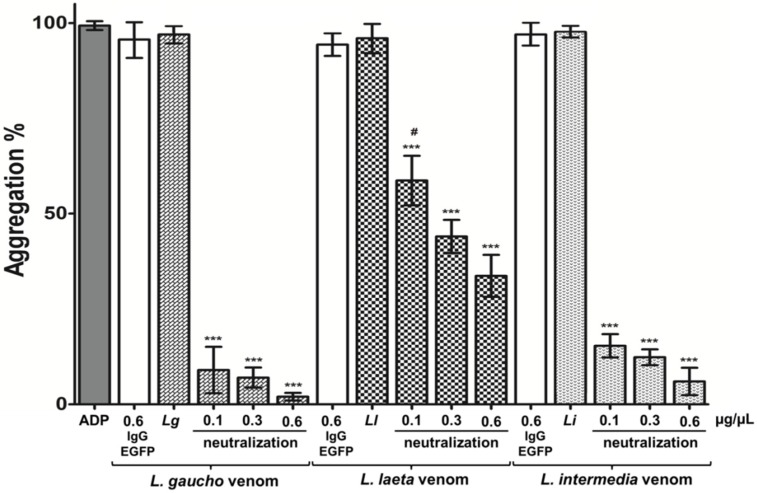
Analysis of platelet aggregation neutralization by anti-LgRec1ALP1. Platelet-rich plasma (PRP) was treated with 7.5 μg of the venoms *L. gaucho* (*Lg*), *L. laeta* (*Ll*), or *L. intermedia* (*Li*) previously incubated or not with 0.1, 0.3, or 0.6 μg/μL of anti-LgRec1ALP1 in a final volume of 100 μL (neutralization). Incubation of *L. gaucho*, *L. laeta*, or *L. intermedia* venoms with 0.6 μg/μL anti-EGFP (IgG-EGFP) were used as negative controls. Platelet aggregation was induced by adding 10 μM of adenosine diphosphate (ADP) in phosphate buffered saline (PBS) as a positive control. Aggregation was monitored by measuring the light transmittance for five minutes by an aggregometer (*n* = 3). Values given are the average ± SEM. Significance was evaluated with an ANOVA one-way with the post-hoc Tukey test; (**) indicates *p* < 0.01, (***) indicates *p* < 0.001. # indicates statistical significance with *p* < 0.05 between samples of the venom’s groups.

The activity of platelet aggregation is one of the main characteristics in *Loxosceles* envenomation. In order to neutralize this activity in vitro, platelet-rich plasma (PRP) was incubated with *L. gaucho*, *L. laeta*, and *L. intermedia* venoms previously pre-incubated or not with 0.1, 0.3, or 0.6 μg/μL of purified anti-LgRec1ALP1. The results show that 0.6 μg/µL of anti-LgRec1ALP1 was effective to neutralize ~100, 94, and 66% of the aggregating activity of *L. gaucho*, *L. intermedia*, and *L. laeta* venoms, respectively (Figure 6). In addition, 0.1 and 0.3 μg/µL of anti-LgRec1ALP1 were also effective to neutralize 91 and 93% of *L. gaucho* venom; 85 and 88% of *L. intermedia* venom and 41 and 56% for *L. laeta* venom, respectively. Platelet aggregation responsiveness was evaluated with 10 mM of adenosine diphosphate (ADP) agonist, and pre-incubation of *L. gaucho*, *L. laeta*, or *L. intermedia* venoms with 0.6 μg/µL of anti-EGFP antibody were used as negative controls (Figure 6, IgG EGFP).

#### 2.4.3. Neutralization of Dermonecrosis and Edema Caused by *Loxosceles* spp. Venoms

**Figure 7:**
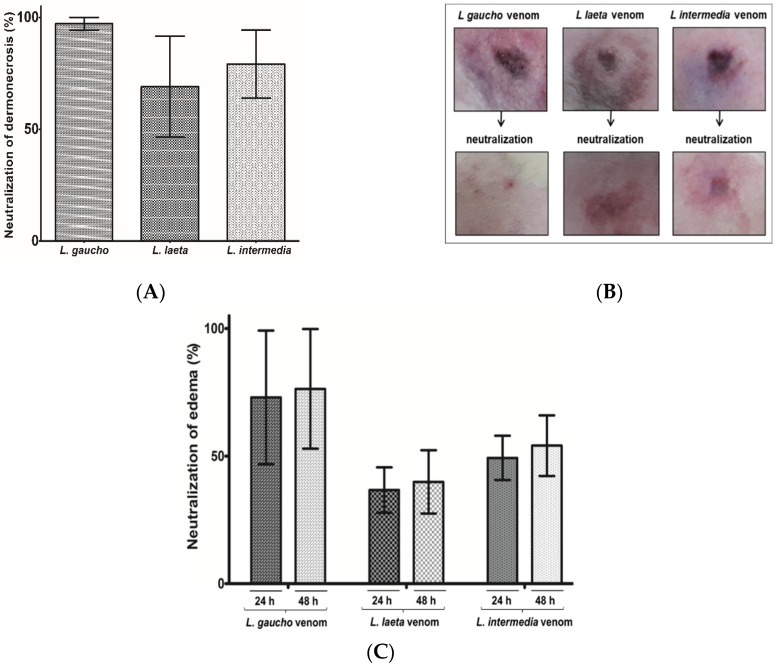
Neutralization of dermonecrosis (**A**) and edema (**C**) induced by *Loxosceles* spp. venoms after incubation with anti-LgRec1ALP1. (**B**) Rabbit’s treated with *Loxosceles* venom or venoms incubated with anti-LgRec1ALP1. To the tests, 6 μg of the venoms *L. gaucho*, *L. laeta*, or *L. intermedia* were pre-incubated with 0.4 μg/µL of anti-LgRec1ALP1 in a final volume of 150 µL for 30 min at 37 °C, centrifuged and the supernatant injected i.d. into the rabbit dorsum. The animals were observed for 24 and 48 h. Size of the lesions was measured by ImageJ software and the results are expressed as the percentage reduction of the size of the lesions. Values given are the average ± SEM (*n* = 2).

Since local reactions such as edema and dermonecrosis are afflictions related to *Loxosceles* spp. envenomation, the neutralizing of this activity by the anti-LgRec1ALP1 were evaluated in rabbits´ skin. For this, 6 μg of the venoms *L. gaucho*, *L. laeta*, or *L. intermedia* were incubated with 0.4 μg/µL of anti-LgRec1ALP1 in a final volume of 150 µL and the area of lesions were measured 24 and 48 h after injection (Figure 7B). As seen in Figure 7A, the anti-LgRec1ALP1 was very effective to abolish all dermonecrosis caused by *L. gaucho* venom, 79% for *L. intermedia*, and 68% *L. laeta* venoms. The edema was also neutralized by the anti-LgRec1ALP1, although in less extent, showing neutralization of 73 and 76% for *L. gaucho*, 37 and 40% for *L. laeta* and 49 and 54% for *L. intermedia* venom in 24 and 48 h, respectively (Figure 7C).

## 3. Discussion

The search for new therapies and strategies for the treatment of people that suffer accidents with venomous animals is increasing every year and therefore it is considered a public health problem. In this sense, spiders of the genus *Loxosceles* spp. are of great medical importance, with several cases reported worldwide [49,50,51,52,53]. In Brazil, these spiders are responsible for thousands of accidents every year (Sistema de Informação de Agravo de Notificação, Ministério da Saúde) and the recommended treatment is the serum therapy [1,27,28,29]. However, due to the limited amount of venom extracted from the *Loxosceles* spp. that is used to produce the antiserum, many studies have been searching for alternatives such as the use of recombinant toxins. In this context, the recombinant phospholipases D (PLDs) or their peptides have been exploited [34,35,36,38,54,55] as these toxins are the main responsible for the symptoms related to the envenoming [11,13,21,22,23]. Nonetheless, antibodies against PLDs alone were not effective to neutralize some venom activities, presumably due to the presence of other toxins that can act synergistically with the PLDs.

Analyzing the transcriptomic profile of *L. gaucho* venom gland, we showed that the PLDs (70.43%) and the astacin-like metalloproteases (ALMPs) (17.58%) accounted for most of the toxin transcripts. Other toxins with lower expression were also found such as insecticidal peptides (TX) (6.21%) with action on Na^+^ channels [56], venom allergens (2.12%) that elicit allergic response similar to other sources such as plant pollens, molds, and foods [57], translationally-controlled tumor protein (TCTP) (0.06%) that has been related to cause edema and vascular permeability [58] serine proteases (2.65%) described to have gelatinolytic activity [59]; serine proteases inhibitors (0.43%), which may be related to coagulation processes and fibrinolysis [39]; phospholipase A2 (0.42%) related to low myotoxic activity at high doses [12] and hyaluronic acid (0.12%), which have shown activity on hyaluronic acid and chondroitin sulfate [11,60]. In agreement with our results, the transcriptome of *L. laeta* [39] and *L. intermedia* venom gland [15] also showed a high level of expression of PLDs and ALMPs.

Because of their proteolytic activities on molecules such as fibronectin [41,42] and fibrinogen [9,44,45], these toxins may work synergistically with other toxins present in the venom, which may explain the local hemorrhage at the bite site, imperfect platelet adhesion and difficulties in wound healing. Therefore, in an effort to develop a new immunogen for raising broadly neutralizing antibodies against these two main toxins from *Loxosceles* venom, in this work we show the construction of a hybrid immunogen, called LgRec1ALP1, that was designed with the hydrophilic regions of the PLD LgRec1 [20] and the metalloprotease LgALP1 highly expressed in the *L. gaucho* venom gland. The rationale was that the hydrophilic regions are more exposed on the toxins surface and some of them might be essential to interact with receptors, therefore, antibodies raised against these regions could confer better neutralization activities.

The hybrid immunogen LgRec1ALP1 was successfully expressed as inclusion bodies and although some refolding protocols such as dialysis, dilution, and adsorption chromatography were performed [61], none of them seemed to work (data not shown). A plausible explanation might be the presence of peptides from astacin-like metalloprotease since most of the recombinant PLDs are soluble, previous work on metalloprotease expression from *L. intermedia* showed to be insoluble [45,62]. However, several other factors may contribute to the inclusion bodies formation [63] and it is very common during overexpression of heterologous genes in *E. coli*, particularly from animal origin. Although the biological activity of the protein in this state is impaired, some studies show that insoluble proteins can successfully be used to produce polyclonal antibodies [64,65,66]. In addition, the inclusion bodies may represent some advantages since they are less vulnerable to degradation and may remain longer in tissues, avoiding their fast clearance, which could, in theory, require fewer boosters or even the necessity of using adjuvants. In fact, some studies have been explored the use of inclusion bodies as a vaccine [67,68,69]. Therefore, after purification and dialysis, the LgRec1ALP1 was used to produce antiserum even in its insoluble state.

Antibodies raised against whole *Loxosceles* venoms have been described to have cross-reactivity among venoms [11,70], which indicates the presence of common epitopes in their toxins. In this regard, the alignment of the hydrophilic peptides of LgRec1ALP1 showed high identity with PLDs and metalloproteases from *L. intermedia* and average identity with these toxins’ counterparts found in *L. laeta* venom (Table 1). Therefore, a cross-reactivity was expected for the anti-LgRec1ALP1. In fact, the ELISA showed that the higher titer of antibodies was against *L. gaucho* venom components, however, it was verified a significant cross-reactivity of this antiserum with *L. laeta* and *L. intermedia* venoms (Figure 4). In addition, Western blot analysis revealed that anti-LgRec1ALP1 was able to recognize PLDs and metalloproteases from all tested venoms, but only a very faint band was revealed for PLD from *L. laeta*, which might be due to the lower identity between *L. gaucho* and *L. laeta* PLDs (Table 1).

As discussed previously, the proteolytic action of ALMPs on some components of the extracellular matrix and fibrinogen [41,45,62] have brought attention to raise protective antibodies against these toxins. Lima and colleagues [71], for example, used the sequences of an ALMPs from *L. intermedia* to compose a chimera protein to raise neutralizing antibodies. However, in this study, the produced antiserum was tested only on *L. intermedia* venom, which used 100 µg of purified IgGs to achieve complete fibrinogenolytic neutralization. Taking into consideration the same amount of venom used in that study, here we showed that the anti-LgRec1ALP1 was more efficient, since 1.5 µg/µL of it was able to completely neutralize the fibrinogen degradation by *L. gaucho* venom and 3.0 µg/µL neutralized 95 and 83% of *L. laeta* and *L. intermedia* venoms, respectively. This result indicates that the identity shared among the LgRec1ALP1 hydrophilic peptides and the ALMPs from all tested venoms was able to raise antibodies with cross-reactivity neutralizing properties.

Platelet aggregation is another effect associated with *Loxosceles* spp. venoms and many studies indicate that this property is related to the PLDs [20,21,23,72,73]. Since there is no report showing the neutralization of this important activity, in this work the effectiveness of anti-LgRec1ALP1 was tested on three *Loxosceles* venom. The results were quite encouraging since the anti-LgRec1ALP1 was able to inhibit 100, 94 and 66% of platelet aggregation caused by *L. gaucho*, *L. intermedia*, and *L. laeta* venoms, respectively.

A very common clinical picture caused by the venom of *Loxosceles* spiders is the development of a notorious necrotic skin ulcer [74,75,76]. Therefore, the efficacy of anti-LgRec1ALP1 was evaluated to inhibit these activities on the rabbit´s skin. The results showed that the anti-LgRec1ALP1 was efficient in totally neutralizing the venom of *L. gaucho*, while this neutralization was around 79 and 68% for *L. intermedia* and *L. laeta* venoms, respectively. These differences in neutralization may be related to differences in the PLDs from the venoms. In fact, all works with antisera against recombinant PLDs demonstrate effectiveness in neutralizing the dermonecrotic action related to the species used to obtain the antiserum [34].

Another characteristic of *Loxosceles* envenomation is the evolution of edema that is difficult to neutralize when only antisera against PLDs are used [35,77], probably due to the contribution of other toxins present in the venoms as well as the evolution of the inflammatory picture [78]. Regardless of other factors that may be involved, the anti-LgRec1ALP1 was able to neutralize 76, 40 and 54% of this activity elicited by *L. gaucho*, *L. laeta*, and *L. intermedia* venoms, respectively. Although the edema was not fully abolished, the anti-LgRec1ALP1 showed to be promising since other studies using antiserum against recombinant PLDs or their peptides showed to be less effective. In this regard, Duarte and colleagues [36] reported that antibodies raised against the PLD LiD1 from *L. laeta* were able to neutralize only 17% of edema caused by this venom. In addition, using antiserum against PLDs peptides from *L. intermedia* and *L. laeta* venoms, Souza and colleagues [38] showed 40% edema neutralization of *L. intermedia* venom. Thus, the results obtained in the in vitro and in vivo tests with the three predominant *Loxosceles* spp. spiders in South America demonstrate the potential application for the constructed hybrid immunogen.

## 4. Conclusions

Taken together, the results shown in this work indicate that the hybrid immunogen LgRec1ALP1 might represent an interesting alternative antigen to produce neutralizing antibodies against the two main toxins present in the *Loxosceles* venom. The LgRec1ALP1 might also be useful to enrich the whole venom so less amount of it would be necessary which in turn would decrease the number of antigens received by the animals during immunization. In addition, this approach may be further extended to other toxins present in the venom to achieve complete neutralization. This approach may also be useful to solve the problem of the limited amount of venom, time-consuming extractions, and animal handling.

## 5. Materials and Methods

### 5.1. Ethics Committees

The procedures involving animals were conducted according to national laws and policies controlled by Butantan Institute Animal Investigation Ethical Committee. Experimental protocol in mice record n^o^ CEUA 8172250816. Experimental protocol in rabbits records n^o^ CEUAIB 886/12. The IBAMA (Brazilian Institute for the Environment and Renewable Natural Resources) provided animal collection permission n^o^ 15383-2, while CGEN (Board of Genetic Heritage Management) provided the license for genetic patrimony access (02001.005110/2008). All manipulation of microorganisms has been developed in biosafety level P2 area, as authorized by CIBio and CTNBio (National Technical Commission on Biosecurity) (Record n^o^ CQB-030/98 de 30/05/2011). All procedures involving human blood were approved by the Ethical Committee in Research from Municipal Secretary of Health of São Paulo, CAAE: 02990818.3.0000.0086.

### 5.2. Animals and Venoms

BALB/c male mice aged 7 to 8 weeks (18–22 g) and New Zealand adult rabbits (3 to 4 kg) were provided by the Butantan Institute Animal Husbandry. All animals received water ad libitum and food under controlled environmental conditions. The venoms were supplied by the Butantan Institute Venoms Center, resuspended in PBS (phosphate buffered saline). For the library of transcripts, 300 wild-type *Loxosceles gaucho* venom glands were collected as previously described [9] and macerated with 2 mL Trizol reagent (Invitrogen^TM^, Thermo Fisher Scientific, Waltham, MA, USA) as recommended by the manufacturer. Subsequently, mRNA purification was performed using Dynabeads^®^ mRNA Purification Kit (Dynal Biotech-Invitrogen^TM^, Thermo Fisher Scientific, Waltham, MA, USA) and the cDNAs synthesized using the cDNA Synthesis System (Roche^®^, Sigma Life Science, Merck Corporation, Darmstadt, Germany) kit, both following manufacturer’s guidelines.

### 5.3. Sequences and Analysis of Sequenced Transcripts

The preparation of cDNA libraries from the mRNA was performed by initial fragmentation of the sample with a ZnCl_2_ solution’s followed by purification of the desired fragments size and synthesis cDNA by cDNA Synthesis System (Roche^®^, Sigma Life Science, Merck Corporation, Darmstadt, Germany) kit, using Roche random primer. For the assembly of the sequences, it was used the 454 GS Junior Roche Life Science software (Branford CT, USA) of the Butantan Institute in the Special Laboratory of Applied Toxinology (LETA). The program used standards parameters except for the values of minimum identity (95%) and minimum length (50 pb). In this assembly an rRNA filter using the rRNA sequences for arachnids available in GenBank. Only the reads that met the criteria of quality and minimum size were used in the assembly to generate the isotigs. The identification of these transcript isotigs was performed using the Blast2GO platform [79], using the blastx algorithm [80] against GenBank nr (non-redundant) database (https://www.blast2go.com/). The hydrophilicity of the toxins was determined by the ProtScale Tool (http://web.expasy.org/protscale/) using the Hopp–Woods scale [46]. The molecular mass of the hybrid immunogen was calculated by the ProtParam Tool program (https://web.expasy.org/protparam/) and the alignments were performed with Clustal W tool (https://npsa-prabi.ibcp.fr/cgi-bin/npsa_automat.pl?page=npsa_clustalw.html). The tridimensional prediction of PLD LgRec1 and LgALP1 was performed by Phyre2 program in an intensive mode setting using the crystal of a phospholipase D (3LRH) from *L. intermedia* and the metalloprotease (3LQ0) from *Astacus astacus* as templates (http://www.sbg.bio.ic.ac.uk/phyre2/html/page.cgi?id=index). The models were visualized by Chimera software (http://www.cgl.ucsf.edu/chimera/download.html).

### 5.4. Construction of the Hybrid Immunogen

To construct the hybrid immunogen, six hydrophilic regions from the recombinant phospholipase D LgRec1: 1–SNSIETDVSFDKQ; 2–KFNDFLKGLRKVTTPGDSK; 3–KLITGFKETLKNEGHEELLEKVGTDFSGNDDISDVQKTYNKAG; 4–LRGLTRVKAAVANRDSGSG; 5–DKRQSTRDTLDAN; 6–PDITVEILNEAAYKKKFRIATYEDNPWET and three hydrophilic regions from the metalloprotease LgALP1: 1–ALFPGDIKKAMRHIEENTCIKFKSRKNEEGYVKIYKGKKES; 2–HEHTRPDRDLYITVHEDNIRPSSKRNYKKT; 3–LTSARYKDSLTDLDIKKINTLYN), were predicted by ProtScale Tool. The nucleotide sequence of each selected region was linked together and optimized for expression in bacteria by Invitrogen™ Gene Synthesis (GeneArt™), Thermo Fisher Scientific, Waltham, MA, USA. This sequence was then cloned into N-terminus of a 6xHis histidine tag between *Bam*HI and *Hind*III sites of pET-28a(+) (Novagen^®^ Merck Corporation, Darmstadt, Germany) and called LgRec1ALP1.

### 5.5. Recombinant LgRec1ALP1 Expression

For expression of the hybrid immunogen LgRec1ALP1, chemically competent *E. coli* BL21 Star™ (DE3) cells (Invitrogen^TM^, Thermo Fisher Scientific, Waltham, MA, USA) were transformed with the pET28a-LgRec1ALP1 construction and a colony grown on plate LB-agar containing 50 μg/mL of kanamycin for 16 h was transferred into liquid LB medium supplemented with 50 μg/mL of kanamycin and grown for 16 h at 30 °C under shaking at 250 rpm. An aliquot of this culture at the 1:50 dilution was added into LB medium supplemented with 50 μg/mL of kanamycin and incubated at 30 °C under agitation of 250 rpm until reaching the logarithmic exponential growth phase (DO 600, ~0.6). At this time, 1 mM of final isopropyl-β-d-thiogalactoside (IPTG) was added in culture and incubated for 4 h at 30 °C. After this period the cells were collected by centrifugation (10,000× *g*) for 15 min at 4 °C and either immediately used or stored frozen at −20 °C.

### 5.6. LgRec1ALP1 Purification

Cells were resuspended in binding buffer with urea 6 M (20 mM de sodium phosphate pH 7.0, 500 mM NaCl and 20 mM of imidazole) and lysed by an ultrasonication intermittently (amplitude of 20% with 3 s pulse and 4 s interval between each pulse) on ice for 90 s with 4 min intervals between each sonication for cooling purposes. This process was repeated five times. The lysate was centrifuged at 10,000 *g* for 10 min at 4 °C and the supernatant containing the solubilized protein was purified by immobilized metal affinity chromatography (IMAC) using 1 mL of Ni Sepharose^®^ 6 Fast Flow GE^®^ resin (Healthcare, Little Chalfont, UK) following the manufacturer’s protocol. LgRec1ALP1 was eluted in elution buffer (20 mM sodium phosphate, 500 mM NaCl and 1 M Imidazole and 6M urea), dialyzed against TBS buffer (20 mM Tris, 150 mM NaCl, pH 7.5) with 3 mM DTT (Dithiothreitol) and analyzed on a 12.5% SDS-PAGE [81] under reducing conditions.

### 5.7. SDS-Polyacrylamide Gel Electrophoresis

Samples were analyzed with constant current of 25 mA on a 12.5% SDS-PAGE containing the same number of bacteria (determined by spectrometry) before and after IPTG induction or 20 μL of purified LgRec1ALP1 in sample buffer (62.5 mM Tris pH 6.8, 10% glycerol, 2% SDS, and 2.5% dithiothreitol) boiled for 5 min. The gels were stained with Coomassie R-250 blue. The molecular mass was estimated by PageRuler™ Prestained Protein Ladder (Thermo Fisher Scientific, Waltham, MA, USA) molecular weight standard.

### 5.8. Quantification of Recombinant Proteins and Venoms

The concentrations of the *L. gaucho*, *L. laeta* and *L. intermedia* venoms and the recombinant PLD LgRec1 and EGFP were determined in duplicate by the bicinchoninic acid method [82] using the Pierce™ BCA Protein Assay Kit (Thermo Fisher Scientific, Waltham, MA, USA) and BSA (Sigma Chemicals, St. Louis, MO, USA) as the standard curve following the manufacturer’s protocol. The hybrid immunogen LgRec1ALP1 and the recombinant ALMP LgALP1, due to their insolubility, had their bands on the SDS-PAGE quantified by the freeware ImageJ, using different concentrations of bovine serum albumin (BSA) as a reference. ImageJ is a Java-based program developed by Wayne Rasband of the National Institute of Health (USA) and is available for download at http://rsb.info.nih.gov/ij/. The version used in this work was downloaded in 15/12/2018 (ImageJ bundled with 64-bit Java 1.8.0_112) using the Windows version [83].

### 5.9. Production of Anti-LgRec1ALP1 in Mice

To obtain polyclonal antibodies anti-LgRec1ALP1, a group of five BALB/c mice were immunized subcutaneously (s.c.) in the base of the tail (0.2 mL/animal) with 10 μg of LgRec1ALP1 in TBS buffer and emulsified in 0.2 mL of Montanide ISA50V. The animals were boosted i.d. 15, 30 and 45 days later with the same dose of antigen with an adjuvant. For the collection of the antiserum, the mice were euthanized in a CO_2_ chamber, whole blood was collected by cardiac puncture and the serum obtained by centrifugation (4 °C, 10 min, 800 *g*). IgGs were purified by affinity chromatography using Protein G Sepharose^TM^ 4 Fast Flow (GE Healthcare, Little Chalfont, UK), following the manufacturer’s protocol. The concentration was determined in duplicate by the bicinchoninic acid method [82] using the Pierce™ BCA Protein Assay Kit (Thermo Fisher Scientific, Waltham, MA, USA) using the BSA (Sigma Chemicals, St. Louis, MO, USA) as the standard curve following the manufacturer’s protocol. Purified mice IgGs against the recombinant enhanced green fluorescent protein (EGFP) were used as a control.

### 5.10. Immunoenzymatic Assay (ELISA)

Polyclonal anti-LgRec1ALP1 antibodies titer was determined by ELISA as described by Theakston and colleagues [84]. Thus, polystyrene plates (Polysorp, NUNC, Roskilde, Denmark) were coated with 5 μg/mL of LgRec1ALP1 diluted in urea 3 M or *L. gaucho*, *L. laeta* or *L. intermedia* venoms diluted in carbonate/bicarbonate buffer (0.05 M, pH 9.6). As a negative control, normal mouse serum was used. The intensity of the reaction was determined by reading the absorbance in ELISA plate reader (Multiskan Spectrophotometer EFLAB, Helsinki, Finland), where titers were determined as the reciprocal of the highest dilution which promotes a reading greater than 0.05 in the length of 492 nm since non-specific reactions should be below this value.

### 5.11. Western Blot Analysis

Samples of the recombinant LgRec1ALP1, LgRec1, LgALP1, EGFP and whole venoms of *L. gaucho*, *L. intermedia*, *L. laeta* were analyzed on a 12.5% SDS-PAGE under reducing conditions. Subsequently, the samples were transferred to nitrocellulose membranes using the Trans-Blot^®^ SD Semi-Dry Transfer Cell (Bio-Rad^®^ Laboratories, Hercules, CA, USA) following the manufacturer’s recommendations. After transfer, the nitrocellulose membranes were stained with Ponceau S^®^ (Merck Millipore Corporation, Darmstadt, Germany) 1:20 to verify the transfer of the proteins. To remove the dye, the membranes were washed with TBS-Tween (20 mM Tris, 150 mM NaCl, 0.05% Tween 20, pH 7.5) until complete removal. Subsequently, the membranes were blocked with incubation buffer (Tris/NaCl, pH 7.5 with 5% milk) for 2 h at room temperature and then washed 3 times with TBS-Tween. Afterward, the membranes were incubated for 2 h with mouse monoclonal anti-polyhistidine antibody (Sigma Life Science, Merck Corporation, Darmstadt, Germany) or anti-LgRec1ALP1 at a 1:1000 dilution in incubation buffer. After, the membranes were washed with TBS-Tween and incubated for 2 h with the peroxidase-labeled anti-mouse IgG (Sigma Life Science, Merck Corporation, Darmstadt, Germany) at a 1:5000 dilution in incubation buffer. Then a new wash cycle was performed and the antigenic components were revealed with 0.05% (*w/v*) 4-chloro-1α-naphthol in 15% (*v/v*) methanol in presence of 0.03% H_2_O_2_ (*v/v*).

### 5.12. Neutralization of Fibrinogen Degradation

For the neutralization tests of the proteolytic activity of the metalloprotease present in the *Loxosceles* sp. venoms, bovine fibrinogen (BF) was dissolved in Tris-HCl buffer (0.05 M HCl, 0.2 M Tris, 0.05 M CaCl_2_, pH 7.4) at the final concentration of 3 µg/µL. In each test, 0.15 µg/µL of the *L. gaucho*, *L. laeta* or *L. intermedia* venoms were pre-incubated with 0.5, 1.5 or 3.0 μg/L of anti-LgRec1ALP1 in a final volume of 20 μL. These reactions were then incubated for 60 min at 37 °C and centrifuged for 5 min at 10,000× *g*. The supernatant of each sample was then mixed with 12 μL of the fibrinogen stock solution and the volume completed to 32 μL. All samples were then incubated for 16 h at 37 °C and analyzed on a 12.5% SDS-PAGE under reducing conditions. BF without venom was used as a control of the reaction and 1,10-ortho-phenanthroline (10 mM) was used to inhibit metalloprotease activity. As a negative control, BF was incubated with 0.15 μg/µL of *L. gaucho*, *L. laeta* or *L. intermedia* venoms previously incubated with 3.0 μg/µL of anti-EGFP. All samples were incubated for 60 min at 37 °C. After this period, all samples were analyzed on a 12.5% SDS-PAGE under reducing conditions and stained with Coomassie blue R-250. The densities of fibrinogen α subunit bands were quantified by the ImageJ freeware and the values were normalized. The experiments were performed in triplicate (*n* = 3) and reported as the mean ± SEM.

### 5.13. Neutralization of Platelet Aggregation

Human blood from healthy volunteers without using medications interfering with platelet activity for at least 10 days prior to testing was collected in 3.8% sodium citrate (1:9). Platelet aggregation using plasma rich in platelets (PRP) was performed as previously described [85]. For the aggregation assay, 7.5 μg of *L. gaucho*, *L. laeta* and *L. intermedia* venoms were pre-incubated or not with 0.1, 0.3 or 0.6 μg/μL of anti-LgRec1ALP1 IgGs in a final volume of 100 μL. The reaction was incubated for 60 min at 37 °C and then centrifuged for 5 min at 10,000 *g* before use. Platelet-poor plasma (PPP) was used as blank and 0.6 μg/μL of IgG anti-EGFP pre-incubated with 7.5 μg of *L. gaucho*, *L. laeta* or *L. intermedia* venoms in a final volume of 100 μL were used as a negative control. The agonist ADP (final concentration of 10 μM) was used as positive control for platelet aggregation. The experiments were performed in triplicate (*n* = 3) on a Chrono-Log Model 490 aggregator (Chrono-Log Corporation, Havertown, PA, USA) and reported as the mean ± SEM.

### 5.14. Neutralization of Dermonecrotic and Edema Activities by the Anti-LgRec1ALP1

To analyze the neutralization of edema and dermonecrotic activities induced by *Loxosceles* spp. venoms, samples of 6 μg of *L. gaucho*, *L. laeta* or *L. intermedia* venoms were incubated with 0.4 μg/μL of anti-LgRec1ALP1 in a final volume of 150 μL for 60 min at 37 °C. Thereafter, the mixtures were centrifuged, and the supernatant was injected i.d. into the rabbit dorsum. The same doses of venoms without antibody were used as a positive control and 0.4 μg/μL of anti-LgRec1ALP1 as a negative control. The animals were observed for 24 and 48 h to analyze the dermonecrosis and edema neutralization. Size of the lesions was measured by ImageJ software and the reduction of the size of the lesions was expressed in percentage. Values are the average ± SEM (*n* = 2).

### 5.15. Statistical Analyses

Statistical analyses were performed using analysis of variance (ANOVA) with the post-hoc Tukey test in the GraphPad Prism 5 software v5.01, 2007. (GraphPad Software, Inc. La Jolla, CA, USA). Significance was considered when *p* < 0.05.

## Figures and Tables

**Table 1:** Amino acids identity analysis of the hydrophilic peptides of LgRec1 from *L. gaucho.*

*L. gaucho*	*L. intermedia*	*L. laeta*
**PLD LgRec1**	**PLD**	**PLD**
**hydrophilic peptides**	**LiRecDT1**	**Smase I**
SNSIETDVSFDKQ	78.6% *	50.0%
KFNDFLKGLRKVTTPGDSK	78.9%	63.1%
KLITGFKETLKNEGHEELLEKVGTDFSGNDDISDVQKTYNKAG	62.7%	55.8%
LLRGLTRVKAAVANRDSGSG	75.0%	40.0%
DKRQSTRDTLDAN	69.2%	38.4%
PDITVEILNEAAYKKKFRIATYEDNPWET	68.9%	51.7%

* Amino acids identity analysis (%) of the hydrophilic peptides of LgRec1 from *L. gaucho* with the PLDs LiRecDT1 (ABA62021) and Smase I (AAM21154) from *L. intermedia* and *L. laeta*, respectively. The identity alignment was obtained using the Clustal W Multiple Sequence Alignment tool.

**Table 2:** Amino acids identity analysis of the hydrophilic peptides of LgALP1 from *L. gaucho.*

*L. gaucho*	*L. intermedia*	*L. laeta*
Metalloprotease LgALP1	Metalloprotease	Metalloprotease
hydrophilic peptides	LALP2	LLAE0237C
ALFPGDIKKAMRHIEENTCIKFKSRKNEEGYVKIYKGKKES	90.4% *	48.7%
HEHTRPDRDLYITVHEDNIRPSSKRNYKKT	90.3%	46.6%
LTSARYKDSLTDLDIKKINTLYN	86.9%	47.8%

* Amino acids identity analysis (%) of the hydrophilic peptides of LgALP1 from *L. gaucho* with the metalloproteases LALP2 (ACV52010) and LLAE0237C (EY188609) from *L. intermedia* and *L. laeta*, respectively. The identity alignment was obtained using the Clustal W Multiple Sequence Alignment tool.

## Supplementary Materials

The following are available online at https://www.mdpi.com/2072-6651/11/2/108/s1, Figure S1: Multiple alignment analysis of deduced amino acid sequences of LgRec1 (AFY98967) from L. gaucho and with the sequence of a phospholipase (PDB: 3RLH) from L. intermedia used as a template to predict the 3D structure of LgRec1; Figure S2: Multiple alignment analysis of deduced amino acid sequences of LgALP1 from *L. gaucho* and the astacin metalloprotease (PDB: 3LQ0) from *Astacus astacus* used as a template to predict the 3D structure of LgALP1.

## References

[1] Hogan C.J., Barbaro K.C., Winkel K. (2004). Loxoscelism: Old obstacles, new directions. Ann. Emerg. Med..

[2] Swanson D.L., Vetter R.S. (2006). Loxoscelism. Clin. Dermatol..

[3] Gremski L.H., Trevisan-Silva D., Ferrer V.P., Matsubara F.H., Meissner G.O., Wille A.C.M., Vuitika L., Dias-Lopes C., Ullah A., de Moraes F.R. (2014). Recent advances in the understanding of brown spider venoms: From the biology of spiders to the molecular mechanisms of toxins. Toxicon.

[4] Málaque C.M., Castro-Valencia J.E., Cardoso J.L., Francca F.O., Barbaro K.C., Fan H.W. (2002). Clinical and epidemiological features of definitive and presumed loxoscelism in São Paulo, Brazil. Rev. Inst. Med. Trop. Sao Paulo.

[5] Pauli I., Puka J., Gubert I.C., Minozzo J.C. (2006). The efficacy of antivenom in loxoscelism treatment. Toxicon.

[6] Chaim O.M., Trevisan-Silva D., Chaves-Moreira D., Wille A.C.M., Ferrer V.P., Matsubara F.H., Mangili O.C., da Silveira R.B., Gremski L.H., Gremski W. (2011). Brown Spider (*Loxosceles genus*) Venom Toxins: Tools for Biological Purposes. Toxins.

[7] Malaque C.M.S., Santoro M.L., Cardoso J.L.C., Conde M.R., Novaes C.T.G., Risk J.Y., Franca F.O.S., de Medeiros C.R., Fan H.W. (2011). Clinical picture and laboratorial evaluation in human loxoscelism. Toxicon.

[8] Da Silva P.H., da Silveira R.B., Appel M.H., Mangili O.C., Gremski W., Veiga S.S. (2004). Brown spiders and loxoscelism. Toxicon.

[9] Da Silveira R.B., Dos Santos Filho J.F., Mangili O.C., Veiga S.S., Gremski W., Nader H.B., Von Dietrich C.P. (2002). Identification of proteases in the extract of venom glands from brown spiders. Toxicon.

[10] Appel M.H., da Silveira R.B., Gremski W., Veiga S.S. (2005). Insights into brown spider and loxoscelism. Invertebr. Surviv. J..

[11] Barbaro K.C., Knysak I., Martins R., Hogan C., Winkel K. (2005). Enzymatic characterization, antigenic cross-reactivity and neutralization of dermonecrotic activity of five *Loxosceles* spider venoms of medical importance in the Americas. Toxicon.

[12] Barbaro K.C., Sousa M.V., Morhy L., Eickstedt V.R.D., Mota I. (1996). Compared chemical properties of dermonecrotic and lethal toxins from spiders of the genus *Loxosceles* (araneae). J. Protein Chem..

[13] Tambourgi D.V., Magnoli F.C., van den Berg C.W., Morgan B.P., de Araujo P.S., Alves E.W., Da Silva W.D. (1998). Sphingomyelinases in the venom of the spider *Loxosceles intermedia* are responsible for both dermonecrosis and complement-dependent hemolysis. Biochem. Biophys. Res. Commun..

[14] Chaim O.M., Sade Y.B., da Silveira R.B., Toma L., Kalapothakis E., Chavez-Olortegui C., Mangili O.C., Gremski W., von Dietrich C.P., Nader H.B. (2006). Brown spider dermonecrotic toxin directly induces nephrotoxicity. Toxicol. Appl. Pharmacol..

[15] Gremski L.H., da Silveira R.B., Chaim O.M., Probst C.M., Ferrer V.P., Nowatzki J., Weinschutz H.C., Madeira H.M., Gremski W., Nader H.B. (2010). A novel expression profile of the *Loxosceles intermedia* spider venomous gland revealed by transcriptome analysis. Mol. Biosyst..

[16] Matsubara F.H., Gremski L.H., Meissner G.O., Constantino Lopes E.S., Gremski W., Senff-Ribeiro A., Chaim O.M., Veiga S.S. (2013). A novel ICK peptide from the *Loxosceles intermedia* (brown spider) venom gland: Cloning, heterologous expression and immunological cross- reactivity approaches. Toxicon.

[17] Manzoni-de-Almeida D., Squaiella-BaptistãO C.C., Lopes P.H., van Den Berg C.W., Tambourgi D.V. (2018). *Loxosceles* venom Sphingomyelinase D activates human blood leukocytes: Role of the complement system. Mol. Immunol..

[18] Barbaro K.C., Cardoso J.L.C., Eickstedt V.R.D., Mota I. (1992). Dermonecrotic and lethal components of loxosceles-gaucho spider venom. Toxicon.

[19] Futrell J.M. (1992). Loxoscelism. Am. J. Med. Sci..

[20] Magalhaes G.S., Caporrino M.C., Della-Casa M.S., Kimura L.F., Prezotto-Neto J.P., Fukuda D.A., Portes J.A., Neves-Ferreira A.G.C., Santoro M.L., Barbaro K.C. (2013). Cloning, expression and characterization of a phospholipase D from *Loxosceles gaucho* venom gland. Biochimie.

[21] Shimokawa-Falcao L., Caporrino M.C., Barbaro K.C., Della-Casa M.S., Magalhaes G.S. (2017). Toxin Fused with SUMO Tag: A New Expression Vector Strategy to Obtain Recombinant Venom Toxins with Easy Tag Removal inside the Bacteria. Toxins.

[22] Kurpiewski G., Forrester L.J., Barrett J.T., Campbell B.J. (1981). platelet-aggregation and sphingomyelinase d activity of a purified toxin from the venom of loxosceles-reclusa. Biochim. Biophys. Acta.

[23] Fukuda D.A., Caporrino M.C., Barbaro K.C., Della-Casa M.S., Faquim-Mauro E.L., Magalhaes G.S. (2017). Recombinant phospholipase D from *Loxosceles gaucho* binds to platelets and promotes phosphatidylserine exposure. Toxins.

[24] Forrester L.J., Barrett J.T., Campbell B.J. (1978). Red blood cell lysis induced by the venom of the brown recluse spider: The role of sphingomyelinase D. Arch. Biochem. Biophys..

[25] Luciano M.N., Da Silva P.H., Chaim O.M., Dos Santos V.L.P., Franco C.R.C., Soares M.F.S., Zanata S.M., Mangili O.C., Gremski W., Veiga S.S. (2004). Experimental Evidence for a Direct Cytotoxicity of *Loxosceles intermedia* (Brown Spider) Venom in Renal Tissue. J. Histochem. Cytochem..

[26] Kusma J., Chaim O.M., Wille A.C.M., Ferrer V.P., Sade Y.B., Donatti L., Gremski W., Mangili O.C., Veiga S.S. (2008). Nephrotoxicity caused by brown spider venom phospholipase-D (dermonecrotic toxin) depends on catalytic activity. Biochimie.

[27] Saude M.D. Manual de Diagnóstico e Tratamento de Acidentes por Animais Peçonhentos. http://bvsms.saude.gov.br/bvs/publicacoes/funasa/manu_peconhentos.pdf.

[28] Guilherme P.C., Fernandes I., Barbaro K.C. (2001). Neutralization of dermonecrotic and lethal activities and differences among 32–35 kDa toxins of medically important *Loxosceles* spider venoms in Brazil revealed by monoclonal antibodies. Toxicon.

[29] Pauli I., Minozzo J.C., Henrique Da Silva P., Chaim O.M., Veiga S.S. (2009). Analysis of therapeutic benefits of antivenin at different time intervals after experimental envenomation in rabbits by venom of the brown spider (*Loxosceles intermedia*). Toxicon.

[30] Dias-Lopes C., Guimarães G., Felicori L., Fernandes P., Emery L., Kalapothakis E., Nguyen C., Molina F., Granier C., Chávez-Olórtegui C. (2010). A protective immune response against lethal, dermonecrotic and hemorrhagic effects of *Loxosceles intermedia* venom elicited by a 27-residue peptide. Toxicon.

[31] Figueiredo L.F.M., Dias-Lopes C., Alvarenga L.M., Mendes T.M., Machado-de-Ávila R.A., McCormack J., Minozzo J.C., Kalapothakis E., Chávez-Olórtegui C. (2014). Innovative immunization protocols using chimeric recombinant protein for the production of polyspecific loxoscelic antivenom in horses. Toxicon.

[32] Karim-Silva S., Moura J.D., Noiray M., Minozzo J.C., Aubrey N., Alvarenga L.M., Billiald P. (2016). Generation of recombinant antibody fragments with toxin-neutralizing potential in loxoscelism. Immunol. Lett..

[33] Tambourgi D.V., Pedrosa M.D.F., van den Berg C.W., Goncalves-de-Andrade R.M., Ferracini M., Paixao-Cavalcante D., Morgan B.P., Rushmere N.K. (2004). Molecular cloning, expression, function and immunoreactivities of members of a gene family of sphingomyelinases from *Loxosceles* venom glands. Mol. Immunol..

[34] de Almeida D.M., Fernandes-Pedrosa M.D., de Andrade R.M.G., Marcelino J.R., Gondo-Higashi H., de Azevedo I., Ho P.L., van den Berg C., Tambourgi D.V. (2008). A new anti-loxoscelic serum produced against recombinant sphingomyelinase D: Results of preclinical trials. Am. J. Trop. Med. Hyg..

[35] Guimarães G., Dias-Lopes C., Duarte C.G., Felicori L., Machado de Avila R.A., Figueiredo L.F.M., de Moura J., Faleiro B.T., Barro J., Flores K. (2013). Biochemical and immunological characteristics of Peruvian *Loxosceles laeta* spider venom: Neutralization of its toxic effects by anti-loxoscelic antivenoms. Toxicon.

[36] Duarte C.G., Bonilla C., Guimarães G., Machado de Avila R.A., Mendes T.M., Silva W., Tintaya B., Yarleque A., Chávez-Olórtegui C. (2015). Anti-loxoscelic horse serum produced against a recombinant dermonecrotic protein of Brazilian *Loxosceles intermedia* spider neutralize lethal effects of Loxosceles laeta venom from Peru. Toxicon.

[37] Felicori L., Fernandes P.B., Giusta M.S., Duarte C.G., Kalapothakis E., Nguyen C., Molina F., Granier C., Chávez-Olórtegui C. (2009). An in vivo protective response against toxic effects of the dermonecrotic protein from *Loxosceles intermedia* spider venom elicited by synthetic epitopes. Vaccine.

[38] Souza N.A., Dias-Lopes C., Matoso Í.H.G., de Oliveira C.F.B., CháVez-Olortegui C.D., Minozzo J.C., Felicori L.F. (2018). Immunoprotection elicited in rabbit by a chimeric protein containing B-cell epitopes of Sphingomyelinases D from *Loxosceles* spp. spiders. Vaccine.

[39] Fernandes-Pedrosa M.d.F., Junqueira-de-Azevedo I.d.L.M., Gonçalves-de-Andrade R.M., Kobashi L.S., Almeida D.D., Ho P.L., Tambourgi D.V. (2008). Transcriptome analysis of *Loxosceles laeta* (Araneae, Sicariidae) spider venomous gland using expressed sequence tags. BMC Genom..

[40] Williamson A.L., Lustigman S., Oksov Y., Deumic V., Plieskatt J., Mendez S., Zhan B., Bottazzi M.E., Hotez P.J., Loukas A. (2006). Ancylostoma caninum MTP-1, an Astacin- Like Metalloprotease Secreted by Infective Hookworm Larvae, Is Involved in Tissue Migration. Infect. Immun..

[41] Feitosa L., Gremski W., Veiga S.S., Elias M.C., Graner E., Mangili O.C., Brentani R.R. (1998). Detection and characterization of metalloproteinases with gelatinolytic, fibronectinolytic and fibrinogenolytic activities in brown spider (*Loxosceles intermedia*) venom. Toxicon.

[42] Veiga S.S., Zanetti V., Braz A., Mangili O.C., Gremski W. (2001). Extracellular matrix molecules as targets for brown spider venom toxins. Braz. J. Med. Biol. Res..

[43] Young A.R., Pincus S.J. (2001). Comparison of enzymatic activity from three species of necrotising arachnids in Australia: *Loxosceles rufescens*, *Badumna insignis* and *Lampona cylindrata*. Toxicon.

[44] Zanetti V.C., da Silveira R.B., Dreyfuss J.L., Haoach J., Mangili O.C., Veiga S.S., Gremski W. (2002). Morphological and biochemical evidence of blood vessel damage and fibrinogenolysis triggered by brown spider venom. Blood Coagul. Fibrinolysis.

[45] Da Silveira R.B., Wille A.C.M., Chaim O.M., Appel M.H., Silva D.T., Franco C.R.C., Toma L., Mangili O.C., Gremski W., Dietrich C.P. (2007). Identification, cloning, expression and functional characterization of an astacin-like metalloprotease toxin from *Loxosceles intermedia* (brown spider) venom. Biochem. J..

[46] Hopp T.P., Woods K.R. (1981). Prediction of protein antigenic determinants from amino acid sequences. Proc. Natl. Acad. Sci. USA.

[47] de Giuseppe P.O., Ullah A., Silva D.T., Gremski L.H., Wille A.C.M., Moreira D.C., Ribeiro A.S., Chaim O.M., Murakami M.T., Veiga S.S. (2011). Structure of a novel class II phospholipase D: Catalytic cleft is modified by a disulphide bridge. Biochem. Biophys. Res. Commun..

[48] Guevara T., Yiallouros I., Kappelhoff R., Bissdorf S., Stöcker W., Gomis-Rüth F.X. (2010). Proenzyme structure and activation of astacin metallopeptidase. J. Biol. Chem..

[49] Nicholson G.M., Graudins A. (2003). Antivenoms for the Treatment of Spider Envenomation. J. Toxicol. Toxin Rev..

[50] Isbister G.K., Fan H.W. (2011). Spider bite. Lancet.

[51] Hubiche T., Delaunay P., del Giudice P. (2013). A case of loxoscelism in southern France. Am. J. Trop. Med. Hyg..

[52] Coutinho I., Rocha S., Ferreira M.E., Vieira R., Cordeiro M.R., Reis J.P. (2014). Cutaneous loxoscelism in Portugal: A rare cause of dermonecrosis. Acta Med. Port..

[53] Morales-Moreno H.J., Carranza-Rodriguez C., Borrego L. (2016). Cutaneous loxoscelism due to *Loxosceles rufescens*. J. Eur. Acad. Dermatol. Venereol..

[54] Olvera A., Ramos-Cerrillo B., Estévez J., Clement H., de Roodt A., Paniagua-Solís J., Vázquez H., Zavaleta A., Salas Arruz M., Stock R.P. (2006). North and South American *Loxosceles* spiders: Development of a polyvalent antivenom with recombinant sphingomyelinases D as antigens. Toxicon.

[55] Fernandes Pedrosa M.d.F., Junqueira de Azevedo I.d.L.M., Gonçalves-de-Andrade R.M., van Den Berg C.W., Ramos C.R.R., Lee Ho P., Tambourgi D.V. (2002). Molecular cloning and expression of a functional dermonecrotic and haemolytic factor from *Loxosceles laeta* venom. Biochem. Biophys. Res. Commun..

[56] De Castro C.S., Silvestre F.G., Araujo S.C., Gabriel de M.Y., Mangili O.C., Cruz I., Chavez-Olortegui C., Kalapothakis E. (2004). Identification and molecular cloning of insecticidal toxins from the venom of the brown spider *Loxosceles intermedia*. Toxicon..

[57] Arlian L.G. (2002). Arthropod allergens and human health. Ann. Rev. Entomol..

[58] Sade Y.B., Boia-Ferreira M., Gremski L.H., da Silveira R.B., Gremski W., Senff-Ribeiro A., Chaim O.M., Veiga S.S. (2012). Molecular cloning, heterologous expression and functional characterization of a novel translationally-controlled tumor protein (TCTP) family member from *Loxosceles intermedia* (brown spider) venom. Int. J. Biochem. Cell Biol..

[59] Veiga S.S., Feitosa L., dos Santos V.L., de Souza G.A., Ribeiro A.S., Mangili O.C., Porcionatto M.A., Nader H.B., Dietrich C.P., Brentani R.R. (2000). Effect of brown spider venom on basement membrane structures. Histochem. J..

[60] Da Silveira R.B., Chaim O.M., Mangili O.C., Gremski W., Dietrich C.P., Nader H.B., Veiga S.S. (2007). Hyaluronidases in *Loxosceles intermedia* (Brown spider) venom are endo-*β*-N-acetyl-d-hexosaminidases hydrolases. Toxicon.

[61] Hiroshi Y., Masaya M. (2014). Refolding Techniques for Recovering Biologically Active Recombinant Proteins from Inclusion Bodies. Biomolecules.

[62] Trevisan-Silva D., Gremski L.H., Chaim O.M., Da Silveira R.B., Meissner G.O., Mangili O.C., Barbaro K.C., Gremski W., Veiga S.S., Senff-Ribeiro A. (2010). Astacin- like metalloproteases are a gene family of toxins present in the venom of different species of the brown spider (genus Loxosceles). Biochimie.

[63] Upadhyay A.K., Herman C., Murmu A., Singh A., Panda A.K. (2012). Kinetics of Inclusion Body Formation and Its Correlation with the Characteristics of Protein Aggregates in *Escherichia coli*. PLoS ONE.

[64] Novo J., Oliveira M., Magalhães G., Morganti L., Raw I., Ho P. (2010). Generation of Polyclonal Antibodies Against Recombinant Human Glucocerebrosidase Produced in *Escherichia coli*. Mol. Biotechnol..

[65] Yang H., Zhang T., Xu K., Lei J., Wang L., Li Z., Zhang Z. (2011). A novel and convenient method to immunize animals: Inclusion bodies from recombinant bacteria as antigen to directly immunize animals. Afr. J. Biotechnol..

[66] Lorch M.S., Collado M.S., Argüelles M.H., Rota R.P., Spinsanti L.I., Lozano M.E., Goñi S.E. (2019). Production of recombinant NS1 protein and its possible use in encephalitic flavivirus differential diagnosis. Protein Expr. Purif..

[67] Jiang X., Xia S., He X., Ma H., Feng Y., Liu Z., Wang W., Tian M., Chen H., Peng F. (2019). Targeting peptide-enhanced antibody and CD11c(+)dendritic cells to inclusion bodies expressing protective antigen against ETEC in mice. FASEB J..

[68] Kesik M., Saczyńska V., Szewczyk B., Płucienniczak A. (2004). Inclusion bodies from recombinant bacteria as a novel system for delivery of vaccine antigen by the oral route. Immunol. Lett..

[69] Wedrychowicz H., Kesik M., Kaliniak M., Kozak-Cieszczyk M., Jedlina-Panasiuk L., Jaros S., Plucienniczak A. (2007). Vaccine potential of inclusion bodies containing cysteine proteinase of Fasciola hepatica in calves and lambs experimentally challenged with metacercariae of the fluke. Vet. Parasitol..

[70] Such D.R., Souza F.N., Meissner G.O., Morgon A.M., Gremski L.H., Ferrer V.P., Trevisan-Silva D., Matsubara F.H., Boia-Ferreira M., Sade Y.B. (2015). Brown spider (*Loxosceles* genus) venom toxins: Evaluation of biological conservation by immune cross-reactivity. Toxicon.

[71] Lima S.d.A., Guerra-Duarte C., Costal-Oliveira F., Mendes T.M., Figueiredo L.F.M., Oliveira D., Machado de Avila R.A., Ferrer V.P., Trevisan-Silva D., Veiga S.S. (2018). Recombinant Protein Containing B-Cell Epitopes of Different Spider Toxins Generates Neutralizing Antibodies in Immunized Rabbits. Front. Immunol..

[72] Tavares F.L., Peichoto M.E., Rangel D.D., Barbaro K.C., Cirillo M.C., Santoro M.L., Sano-Martins I.S. (2011). *Loxosceles gaucho* spider venom and its sphingomyelinase fraction trigger the main functions of human and rabbit platelets. Hum. Exp. Toxicol..

[73] Appel M.H., Da Silveira R.B., Chaim O.M., Paludo K.S., Silva D.T., Chaves D.M., Da Silva P.H., Mangili O.C., Senff-Ribeiro A., Gremski W. (2008). Identification, cloning and functional characterization of a novel dermonecrotic toxin (phospholipase D) from brown spider (*Loxosceles intermedia*) venom. Biochim. Biophys. Acta.

[74] Pezzi M., Giglio A.M., Scozzafava A., Filippelli O., Serafino G., Verre M. (2016). Spider Bite: A Rare Case of Acute Necrotic Arachnidism with Rapid and Fatal Evolution. Case Rep. Emerg. Med..

[75] Mariutti R.B., Chaves-Moreira D., Vuitika L., Caruso Í.P., Coronado M.A., Azevedo V.A., Murakami M.T., Veiga S.S., Arni R.K. (2017). Bacterial and Arachnid Sphingomyelinases D: Comparison of Biophysical and Pathological Activities. J. Cell. Biochem..

[76] Chaves-Moreira D., De Moraes F.R., Caruso Í.P., Chaim O.M., Senff-Ribeiro A., Ullah A., Da Silva L.S., Chahine J., Arni R.K., Veiga S.S. (2017). Potential Implications for Designing Drugs Against the Brown Spider Venom Phospholipase-D. J. Cell. Biochem..

[77] Mendes T.M., Oliveira D., Figueiredo L.F.M., Machado-de-Avila R.A., Duarte C.G., Dias-Lopes C., Guimaraes G., Felicori L., Minozzo J.C., Chavez-Olortegui C. (2013). Generation and characterization of a recombinant chimeric protein (rCpLi) consisting of B-cell epitopes of a dermonecrotic protein from *Loxosceles intermedia* spider venom. Vaccine.

[78] Ribeiro M.F., Oliveira F.L., Monteiro-Machado M., Cardoso P.F., Guilarducci-Ferraz V.V.C., Melo P.A., Souza C.M.V., Calil-Elias S. (2015). Pattern of inflammatory response to *Loxosceles intermedia* venom in distinct mouse strains: A key element to understand skin lesions and dermonecrosis by poisoning. Toxicon.

[79] Conesa A., Götz S., García-Gómez J.M., Terol J., Talón M., Robles M. (2005). Blast2GO: A universal tool for annotation, visualization and analysis in functional genomics research. Bioinformatics.

[80] Altschul S.F., Madden T.L., Schäffer A.A., Zhang J., Zhang Z., Miller W., Lipman D.J. (1997). Gapped BLAST and PSI-BLAST: A new generation of protein database search programs. Nucleic Acids Res..

[81] Laemmli U.K. (1970). Cleavage of Structural Proteins during the Assembly of the Head of Bacteriophage T4. Nature.

[82] Smith P.K., Krohn R.I., Hermanson G.T., Mallia A.K., Gartner F.H., Provenzano M.D., Fujimoto E.K., Goeke N.M., Olson B.J., Klenk D.C. (1985). Measurement of protein using bicinchoninic acid. Anal. Biochem..

[83] ImageJ, U.S. imagej.nih.gov/ij/.

[84] Theakston R.D.G., Reid H.A. (1979). Enzyme-linked Immunosorbent Assay (ELISA) in assessing antivenom potency. Toxicon.

[85] Santoro M.L., Sousa-e-Silva M.C.C., Goncalves L.R.C., Almeida-Santos S.M., Cardoso D.F., Laporta-Ferreira I.L., Saiki M., Peres C.A., Sano-Martins I.S. (1999). Comparison of the biological activities in venoms from three subspecies of the South American rattlesnake (*Crotalus durissus terrificus, C. durissus cascavella and C. durissus collilineatus*). Comp. Biochem. Physiol. C: Pharmacol. Toxicol. Endocrinol..

