# Supplementary material for: Design and Production of a Recombinant Hybrid Toxin to Raise Protective Antibodies against Loxosceles Spider Venom
Source: Toxins (Basel). 2019 Feb 12;11(2):108. doi: 10.3390/toxins11020108 (PMC6409891; doi:10.3390/toxins11020108)
Supplement: Supplementary file 1 [file toxins-11-00108-s001.zip › Figure S2.pdf]

A

```
                       10        20        30        40        50        60        70
                        |         |         |         |         |         |         |
LgALP1         NAISFENMRWPNATIVYKLAGIYALFPGDIKKAMRHIEENTCIKFKSRKNEEGYVKIYKGKKESCFADIG
Astacin_3LQ0   AAILGDEYLWSGGVIPYTFAGVSGADQSAILSGMQELEEKTCIRFVPRTTESDYVEIFTSG-SGCWSYVG
                **  ::  *    * * :**:       *   *: :**:***:*  *  *  **:*:      *:: :*

                       80        90       100       110       120       130       140
                        |         |         |         |         |         |         |
LgALP1         YFAKEQRLSLGS-GCKIFGRILHEMGHTIGLFHEHTRPDRDLYITVHEDNIRPSSKRNYKKTPAAMTRVI
Astacin_3LQ0   RISGAQQVSLQANGCVYHGTILHALMHAIGFYHEHTRMDRDNYVTINYQNVDPSMTSNFDIDTYSR-YVG
                ::  *::** : **   * *** : *:**::***** *** *:*:: :*: **   *:     :   *

                      150       160       170       180       190       200
                        |         |         |         |         |         |
LgALP1         GPFDYHSIMIYGENAGSIDPMHLKTMEANTPGITLTSARYKDSLTDLDIKKINTLYNCPGKDKFT
Astacin_3LQ0   EDYQYYSIMHYGKYSFSIQWGVLETIVPLQNGIDLTDPYDKAHMLQTDANQINNLYTNECSLR--
                 ::*:*** **: : **:   *:*:     ** **    *  : : * ::** **.     :
```

B

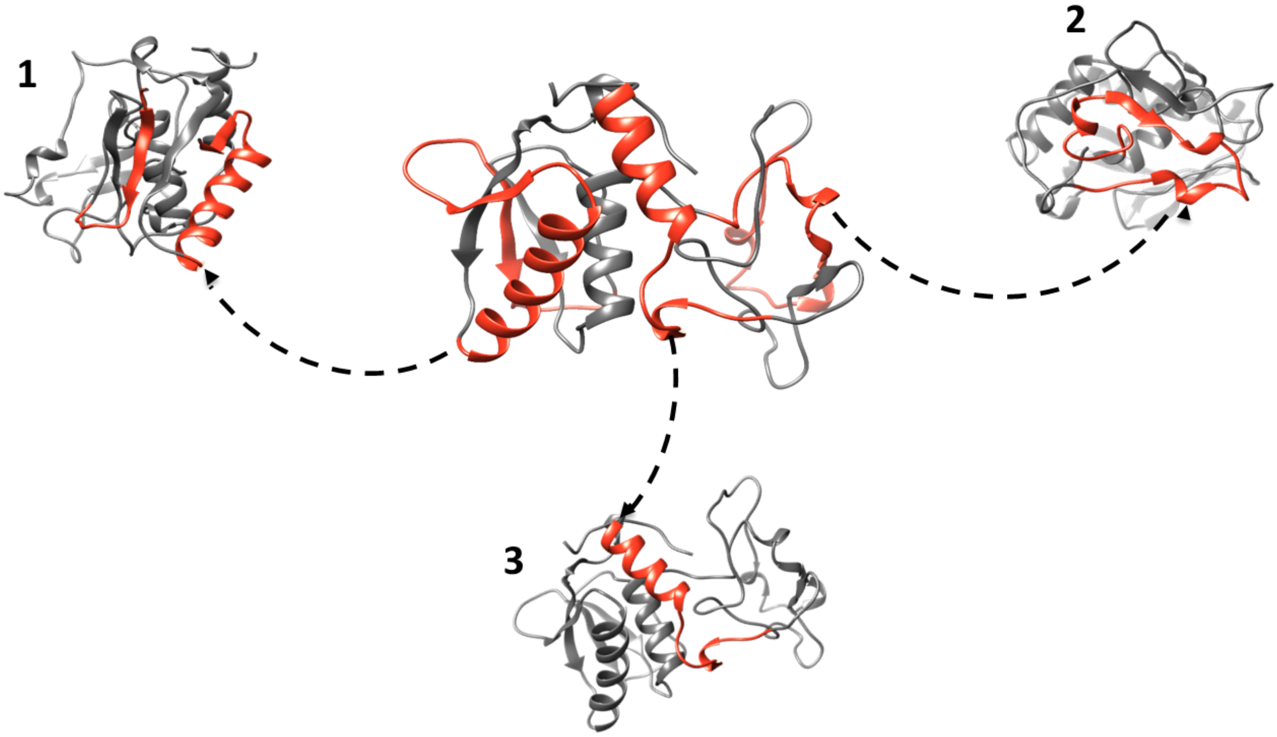

Figure S2: (A) Multiple alignment analysis of deduced amino acid sequences of LgALP1 from *L. gaucho* and the astacin metalloprotease (PDB: 3LQ0) from *Astacus astacus* used as a template to predict the 3D structure of LgALP1. The hydrophilic regions of LgALP1 used to construct the hybrid immunogen are underlined. **The cysteines and the astacin family motifs, such as the catalytic site (HEXXHXXGXXHE) and the Met-turn (MXY), are indicated in boldface**. Identical amino acids are indicated by (*), while conservative substitutions are indicated by (:). (B) Predicted tridimensional structure of ALMP LgALP1 showing the hydrophilic regions. The central picture shows highlighted in orange the three hydrophilic regions of LgALP1 chosen to construct the hybrid immunogen LgRec1ALP1. Each hydrophilic region is shown around the central picture. (1-ALFPGDIKKAMRHIEENTCIKFKSRKNEEGYVKIYKGKKES; 2-HEHTRPDRDLYITVHEDNIRPSSKRNYKKT; 3-LTSARYKDSLTDLDIKKINTLYN.
