# Supplementary material for: Design and Production of a Recombinant Hybrid Toxin to Raise Protective Antibodies against Loxosceles Spider Venom
Source: Toxins (Basel). 2019 Feb 12;11(2):108. doi: 10.3390/toxins11020108 (PMC6409891; doi:10.3390/toxins11020108)
Supplement: Supplementary file 1 [file toxins-11-00108-s001.zip › Figure S1.pdf]

A

```
                                 10        20        30        40        50        60
                                  |         |         |         |         |         |
LgRec1                   ADNRRPIWVMGHMVNSLAQIDEFVGLGSNSIETDVSFDKQANPEYTYHGIPCDCGRACLH
phospholipase_D_3RLH     AGNRRPIWIMGHMVNAIGQIDEFVNLGANSIETDVSFDDNANPEYTYHGIPCDCGRNCKK
                         * ******:******:: ****** **:********** :**************** * :

                                 70        80        90       100       110       120
                                  |         |         |         |         |         |
LgRec1                   STKFNDFLKGLRKVTTPGDSKYLEKLILVVFDLKTGSLYDNQAYDAGTKLAKNLLQHYWN
phospholipase_D_3RLH     YENFNDFLKGLRSATTPGNSKYQEKLVLVVFDLKTGSLYDNQANDAGKKLAKNLLQHYWN
                           :*********  ****:*** ***:**************** *** ************

                                130       140       150       160       170       180
                                  |         |         |         |         |         |
LgRec1                   NGNNGGRAYIILSIPNLNHYKLITGFKETLKNEGHEELLEKVGTDFSGNDDISDVQKTYN
phospholipase_D_3RLH     NGNNGGRAYIVLSIPDLNHYPLIKGFKDQLTKDGHPELMDKVGHDFSGNDDIGDVGKAYK
                         **********:****:**** ** ***: * ::** **::*** ******** ** *:*:

                                190       200       210       220       230       240
                                  |         |         |         |         |         |
LgRec1                   KAGVTGHVWQSDGITNCLLRGLTRVKAAVANRDSGSGIINKVYYWTVDKRQSTRDTLDAN
phospholipase_D_3RLH     KAGITGHIWQSDGITNCLPRGLSRVNAAVANRDSANGFINKVYYWTVDKRSTTRDALDAG
                         ***:***:********** ***:**:********  *:************ :***:***

                                250       260       270       280
                                  |         |         |         |
LgRec1                   VDGIMTNYPDITVEILNEAAYKKKFRIATYEDNPWETFKG
phospholipase_D_3RLH     VDGIMTNYPDVITDVLNEAAYKKKFRVATYDDNPWVTFKK
                         **********:  ::***********:***:**** *** 
```

B

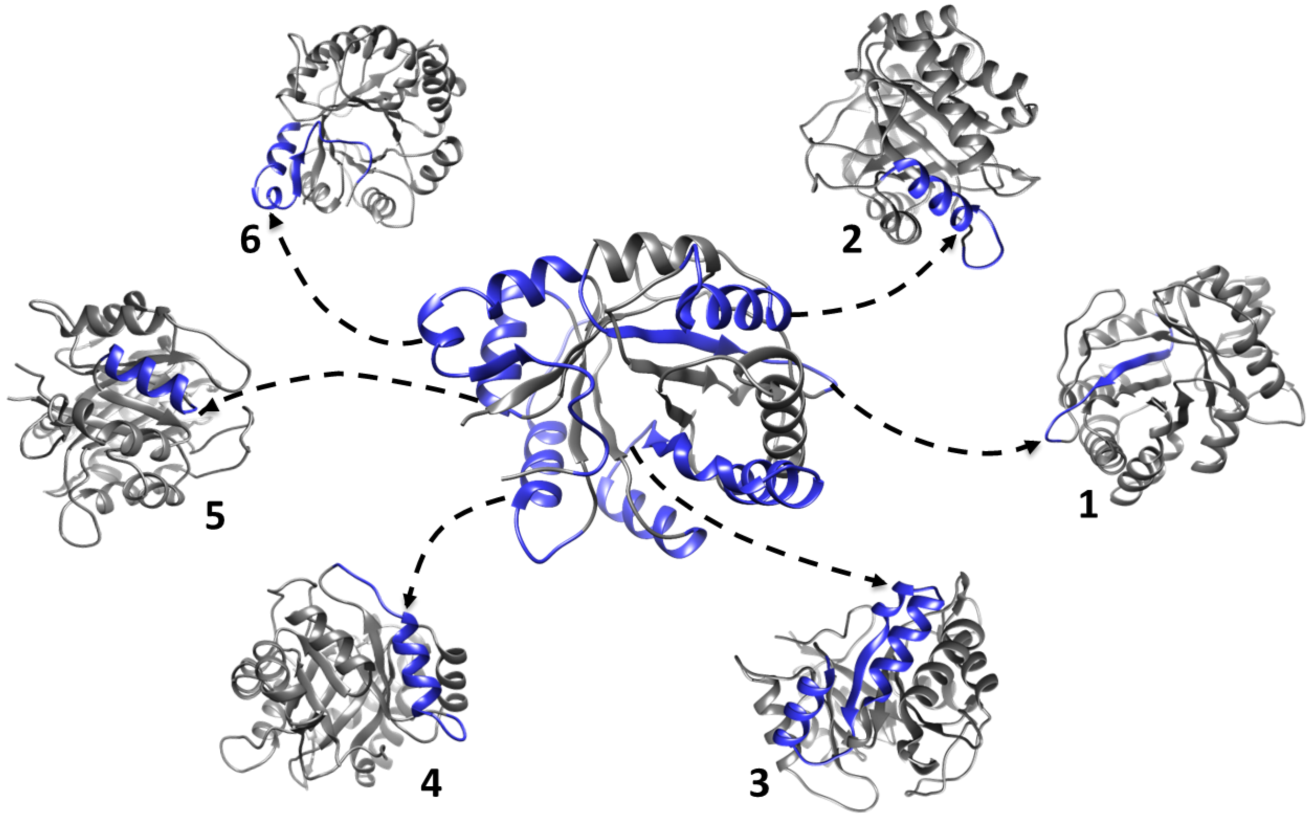

Figure S1: (A) Multiple alignment analysis of deduced amino acid sequences of LgRec1 (AFY98967) from *L. gaucho* and with the sequence of a phospholipase (PDB: 3RLH) from *L. intermedia* used as a template to predict the 3D structure of LgRec1. The hydrophilic regions of LgRec1 used to construct the hybrid immunogen are underlined. **The cysteines and the residues that are involved in the catalysis are shown in boldface**. Identical amino acids and conservative substitutions are indicated by (*) and (:), respectively. (B) Predicted tridimensional structure of PLD LgRec1 showing the hydrophilic regions. The central picture shows highlighted in blue the six hydrophilic regions of LgRec1 chosen to construct the hybrid immunogen LgRec1ALP1. Each hydrophilic region is shown around the central picture. (1-SNSIETDVSFDKQ; 2-KFNDFLKGLRKVTTPGDSK; 3-KLI TGFKETLKNEGHEELLEKVGTDFSGNDDISDVQKTYNKAG; 4-LLRGLTRVKAAVANRDSGSG; 5-DKRQSTRDTLDAN; 6-PDITVEILNEAAYKKKFRIATYEDNPWET.
